# Young sanctuary-living chimpanzees produce more communicative expressions with artificial objects than with natural objects

**DOI:** 10.1098/rsos.240632

**Published:** 2024-10-23

**Authors:** Violet Gibson, Derry Taylor, Sarah Salphati, Eszter Somogyi, Iris Nomikou, Marina Davila-Ross

**Affiliations:** ^1^ School of Psychology, Sport and Health Sciences, University of Portsmouth, King Henry Building, King Henry 1st Street, Portsmouth PO1 2DY, UK; ^2^ Faculty of Sport, Health, and Social Science, Solent Southampton University, East Park Terrace, Southampton SO14 0YN, UK; ^3^ Institute of Biology, University of Neuchâtel, rue Emile-Argand 11, Neuchâtel 2000, Switzerland

**Keywords:** object use, language evolution, vocalizations, primate communication, tool use

## Abstract

In humans, interactions with objects are often embedded in communicative exchanges. Objects offer unique affordances to explore, carry functions and hold cultural relevance, which can shape children’s interactions and communication. Research indicates that the use of artificial objects, such as certain toys, helps promote pre-linguistic communication, consequently impacting language development. Given that chimpanzees use objects extensively compared to other great apes, and considering the differences between chimpanzees and bonobos in intrinsic motivation for tool use and the extended developmental period during which they learn to use objects, it is reasonable to expect that objects may influence chimpanzees’ communication. Here, we examined interactions of 31 immature sanctuary-living chimpanzees with non-novel artificial and natural objects and tested their vocal and facial expressions, applying methods previously designed for children. Our results showed an increase in these expressions associated with artificial objects. These findings provide the first empirical evidence that chimpanzee communicative expressions may be influenced by inherent properties of objects, potentially promoting varied communication, comparable to the impact distinctive objects have on pre-linguistic children. By exploring this connection between object-centric interactions and communication, this study reveals deep phylogenetic roots where objects may have shaped great ape communication and possibly evolutionary foundations of language.

## 1. Introduction

Empirical research has revealed that the production of communicative expressions in human infants may be positively influenced by objects (e.g. [1,2]). Specifically, the use of certain artificial objects, particularly traditional toys, has been shown to elicit more vocalizations and facial displays compared to interactions with electronic toys [3–5]. Furthermore, a recent study compared natural objects (e.g. sticks, leaves, stones, etc.) with toys and household items and found that human infants vocalized significantly more with protophones (speech-like vocalizations) when using artificial objects than the natural ones during interactions with carers [6]. The findings further showed that such an increase in communicative expressions was most likely not induced by the carer’s behaviors but it was instead attributed to the object features. Similar relevance of object features was also observed during infant–carer object transfers, where object properties impacted child’s collaborative behaviors [7]. These properties may include a toy that is, for instance, of particular preference to the child, has symbolic sounds associated with it or one that is categorized by a carer as something that can be shared. Some argue that the early flexibility in infant vocalizations can be partly attributed to the flexibility derived from their increasing ability to manipulate objects, which itself is driven by the infants’ own interest in objects [8,9]. These empirical accounts emphasize the potential role of artificial objects, specifically those with features that generate interest and motivate infants’ communicative expressions. This is in line with some evolutionary accounts of language origins, specifically that vocal language was adapted as a way of sharing and requesting situational information, including information about physical objects as seen in tool making [10]. Nonetheless, our knowledge is limited about the extent to which objects may promote communicative behaviors in young apes, particularly beyond practical tool using applications. By exploring social object use, this study is likely to provide more insight into the relationship between objects and communication development from an evolutionary perspective.

Indeed, our understanding of ape communication involving artificial objects is limited largely by the absence of complex tools or manufactured objects in wild habitats. In light of this issue, we examined sanctuary-living chimpanzees and their social interactions involving natural materials and manufactured (artificial) objects. In terms of other exploratory considerations, this approach attempts to also offer further insight into anthropogenic influences on chimpanzee communication, particularly the impact of artificial objects on their behavior and its implications. Understanding the evolutionary aspects of object use may provide key information for evaluating the origins of human language [11]. In that respect, the present work set out to examine the communicative expressions of infant and juvenile chimpanzees when using natural and artificial objects in social interactions, mirroring the developmental study of Gibson *et al*. [6]. Chimpanzees show the most proficient and diverse range of tool use among the great apes [12], with intrinsic predispositions towards manipulating objects observed early in development [13]. They are also known to incorporate objects in their social interactions [14], but the extent to which their behavior is influenced by the objects themselves is not well understood. To investigate further the potential parallels in human and chimpanzee communication early in ontogeny, it is necessary to examine the behavior of immature chimpanzees, whose object knowledge is thought to still expand with age [15,16].

Current comparative evidence on how specific object types may relate to the occurrence of facial expressions and vocalizations in chimpanzees is quite limited. The results of existing studies, however, provide some indication that certain events related to objects may prompt vocal signalling, as seen in, for instance, primate food or alarm calls [17–20]. Nonhuman primates have also been observed to use objects as tools to modify or enhance their vocalizations. Examples include gibbons slamming enclosure doors to acoustically accentuate their vocalizations [21] or orangutans using leaves to alter the frequency of their kiss squeak sounds [22]. This illustrates that specific object-related events can influence vocal expressions in nonhuman primates, which points to a direct link between object use and the variation of their communicative behaviours.

This supposed object effect also appears to influence attention-getting behaviours, where physical items can be used to achieve individual goals. For instance, young chimpanzees may throw objects to draw attention to their pout facial expressions, aiding nursing requests [23], or to initiate play with peers [24]. This suggests that even at a young age, chimpanzees can use objects to meet their needs, such as securing care or initiating social interactions. Older chimpanzees may throw objects during displays to enhance their impact by creating noise and attracting attention, without the need for a direct physical confrontation [25]. This behaviour highlights their ability to use objects as tools within a social environment, though their use tends to primarily focus on achieving immediate goals.

Yet, despite this apparent relevance of objects for primate communication and apart from referential pointing where object properties are a requisite that motivates the behaviour [26,27], the effect of object features is rarely examined, even though certain object properties (e.g. rattling noise or depositing food) are known to influence primates’ social behaviours including play and allogrooming [28–30]. Thus, it seems that material objects, possibly due to their perceived significance or appeal, whether arising from their use in social interactions, as practical tools, or as representations of salient features in the chimpanzee environment, may represent specific situational circumstances that prompt communicative expressiveness in nonhuman primates.

The majority of what is known about communicative object use in great apes comes from the gesture literature, which revealed that approximately 10 to 20% of gestural repertoires feature actions with objects, such as object hitting, throwing or waving [31–33]. More dedicated accounts of nonvocal, object-based communication focused on leaf-modifying gestures where variation and flexibility were thought to derive from social learning [34]. Meanwhile, in an attempt to depart from gestural communication, Gibson *et al*. [14] outlined an important observation that chimpanzees use objects in their broader social interactions and do so in targeted ways to communicate with conspecifics. In fact, the said repertoire, featuring 24 distinct object actions, offers further evidence for the chimpanzees’ extensive propensity to integrate objects into their social interactions and the role of objects in primate communication. Interestingly, the authors found the propensity to incorporate objects into communicative actions was modality dependent, with object use being more likely to accompany communication in the visual modality. Such selective object use implies a communicative function potentially revolving around joint attention, however, the way objects function in primate communication appears to differ notably from their role in the interactions of human children.

In children, objects are often central to shared intentionality, being used in joint activities, cooperative tasks and symbolic play [35,36]. These include collaborative problem solving, interactions that require the understanding of others’ intentions and social learning [37]. Chimpanzees also understand others’ goals and intentions within cooperative problem-solving tasks [38], where gaze behaviours play an important communicative role. Chimpanzees have been shown to exhibit gaze-following behaviours [39], social referencing [40] and communicative gaze-marking of external objects [41], but in contrast to humans, explicit attempts to direct others’ attention declaratively remain extremely rare (but see [27]). We may, therefore, expect gaze to play a communicative role in object interactions, with objects increasing communicative behaviors. However, in contrast to humans, although chimpanzees use objects socially, they are less likely to engage in activities involving shared goals or intentions with others compared to humans [35]. Their interactions with objects are more individually motivated, focusing on exploration and simple social play [31] rather than symbolic communication or cooperation. These differences in object use may lead to communicative variations, with chimpanzees’ communication focusing more on the immediate functional use of objects [42], being produced at lower rates in comparison to humans, less explicitly in comparison to humans and with objects primarily used as tools for individual goals such as attracting attention (e.g. during play, displays and exploration) [31].

It is important to note, however, that the types of actions with objects may vary with one’s experience that is shaped by routine activities [43] and social influences [44]. Nonhuman primates are sensitive to object features [45,46], and may develop object relations based on functional associations [43] and their own familiarity [45]. For instance, young primates learn how the physical properties of stones (e.g. their size) may afford certain actions (e.g. banging) [15,47], which is instrumental for later nut-cracking [48]. Tool use also changes stick manipulations in chimpanzees replacing initial exploratory actions with goal-directed behaviors [16]. This emphasizes the importance of exposure, where daily experiences allow for practical properties, affordances and complementary associations to be learned [43,49,50]. In that regard, interactions with artificial objects are likely to differ from those with natural materials. It is important to note that the artificial objects examined in the present study were familiar objects that stayed in the chimpanzee enclosures for a longer time period, so they were not novel to the study subjects. This level of familiarity, thus, gives us the opportunity in this study to examine if the distinctive visual features of artificial objects, for which chimpanzees were found to display a preference [51], may be sufficient to elicit more interest and communicative behaviours. Indeed, the attention and communication these objects may generate could be attributed to the inherent properties of the objects, rather than their novelty. To better understand how objects shape communication in early chimpanzee ontogeny, as done in the human infant literature [4–6], it is important to evaluate which factors may affect their communicative expressions and whether these may vary depending on objects that carry the distinctive features of the object.

Object features, such as one’s familiarity with an object, may also influence social gazing. For instance, interactions with novel objects often lead to more visual checking with others to alleviate uncertainty [40,52–54]. Among great apes, young chimpanzees spend considerable time observing adults, with more experienced individuals serving as important resources of information related to object use and practical tool applications [44,55]. As a result, social gazing, which is an important aspect of nonvocal communication, can indicate and reinforce knowledge related to objects [52]. However, visual attention can also be drawn by other factors, such as the distinct properties of the objects themselves. Features such as colour [56] and shape [57] may attract attention and prompt communicative behaviours, independent of novelty. It is, therefore, important to explore how specific types of objects, such as natural and artificial ones which carry distinctive visual properties, might influence social gazing among young chimpanzees, and how different object types may facilitate or inhibit communication in early chimpanzee ontogeny.

The main objective of the current research was to (i) compare the communicative behaviours of immature chimpanzees when interacting with natural and artificial objects and (ii) assess whether the pattern varies with their age. We studied these behaviours in 31 immature sanctuary-living chimpanzees living at Chimfunshi Wildlife Orphanage (CWO), Zambia. To systematically examine social interactions involving objects, we assessed object actions based on a previously defined repertoire [14]. We tested our hypothesis that chimpanzees would produce more facial expressions and vocalizations during interactions with more visually distinctive artificial objects than with natural objects. Artificial objects used in this study are considered to carry more social and visual appeal than natural objects, and in turn, are likely to promote more social interactions and communicative behaviours. Importantly, the examined chimpanzees were already relatively accustomed to interacting with similar artificial objects, which were not novel but rather common types of artificial objects that had previously been present in their enclosures. These objects featured signs of wear and tear from prior handling, indicating they were not unfamiliar stimuli likely to provoke heightened communicative behaviours due to novelty.

In addition, social gazing was also examined with respect to the object type. Social gazing in nonhuman primates has been previously discussed in terms of observational learning (e.g. related to tool use) [55,58] and sharing attention or interest about certain objects, as seen in, for instance, object showing where responding with object-directed gazing leads to simultaneous attention [27]. We hypothesized that chimpanzees would produce more social gazing during interactions with artificial objects than with natural objects. To support the interpretation of our data, object preference was examined by assessing chimpanzees’ object choices when in the presence of both natural and artificial object options [51,59]. In addition, the effect of familiarity with artificial objects was also explored, by comparing interactions that featured the same artificial object across two observation points. Finally, the behaviors of social partners were also assessed, including their responses and object retrieval.

Together, this research contributes to our collective understanding related to the unique way in which social object use may help promote the development of communicative expressions in chimpanzee ontogeny and provide important insights into the significance of object features that could be deeply rooted in primate lineage. Unlike previous research, which often centres around novelty and practical tool use in adult apes, we explored how objects influence early communication and by extension, the way in which specific object features may help promote the socio-cognitive development in primates.

## 2. Material and methods

### 2.1. Subjects and study site

The subjects were 31 immature chimpanzees living in four sanctuary colonies (*N*_Colony1_ = 44, *N*_Colony2_ = 24, *N*_Colony3_ = 14, *N*_Colony4_ = 12 during the main recording period, i.e. in 2018) at the Chimfunshi Wildlife Orphanage (CWO), Zambia. The sample consisted of 13 infants (up to 4 years old; *F* = 6, *M* = 7, mean age = 1.59 ± s.d. = 1.09) and 18 juveniles (4–9 years old; *F* = 8, *M* = 10, mean age = 6.44 ± s.d. = 1.26) [60] (see table 1 for a sample breakdown). All subjects were born and raised at CWO and reared by their mothers within the colonies.

**Table 1.** Overall sample composition with age group, age, sex and colony membership (*n* = 31).

age group	age (year)	subjects	sex	colony
**infants**				
	<1	3	males (2), females (1)	1, 2
	1	2	males (1), females (1)	1, 2
	2	5	males (3), females (2)	1, 2
	3	3	males (2), females (1)	2
**juveniles**				
	5	5	males (3), females (2)	2, 3, 4
	6	5	males (3), females (2)	1, 2
	7	5	males (3), females (2)	1, 2, 4
	8	1	females (1)	2
	9	2	males (1), females (1)	2, 3

Colonies at CWO are composed of wild-born and sanctuary-born chimpanzees belonging to two chimpanzee sub-species including *Pan troglodytes schweinfurthii* and *Pan troglodytes troglodytes*. The sub-species composition across all colonies was estimated to be 42–65% P. *troglodytes schweinfurthii* and 31–42% *P. troglodytes* in 2011 [61], since which there has been no new addition of wild-born chimpanzees. Currently, wild-born chimpanzees comprise approximately one-third of the chimpanzees housed at CWO, of which 24 originated from countries within the chimpanzee species range (e.g. Uganda, Tanzania, and Rwanda). CWO colonies feature a multi-male, multi-female composition with opportunities to form fission–fusion social dynamics.

CWO is located in the Copperbelt Province of Zambia, in a miombo woodland forest ecologically comparable to that of some wild chimpanzees’ environments [61]. The chimpanzees live in one of four semi-wild, fenced enclosures ranging in size between 25 (colony 4) and 77 (colony 1) hectares. The semi-wild enclosures aim to replicate the natural conditions of the wild habitats while still being within a controlled, managed setting. The enclosures are considerably larger than traditional zoo enclosures, which allows the chimpanzees to exhibit natural behaviours (e.g. foraging, climbing and socializing) over a wide area. The larger social groups are also more representative of chimpanzees’ wild-living communities, allowing for complex interactions and the development of social hierarchies. Unlike traditional zoo enclosures, which are smaller and less environmentally complex, semi-wild settings feature varied terrain and natural vegetation, overall prioritizing the animals’ autonomy and natural lifestyles.

The large groups housed in each enclosure offer immature chimpanzees daily opportunities to interact with peers and adult conspecifics [60]. In addition to social interactions, each group has comparable access to a wide range of natural objects (e.g. branches, sticks, rocks, etc.) and permanent artificial fixtures (e.g. fences, metal window bars, drinking fountains, etc.). Though chimpanzees are rarely provided with artificial enrichment, object availability can vary between colonies due to artificial objects occasionally landing in the enclosures (e.g. plastic bags, drinking bottles, fabric items, etc.). Additionally, artificial objects (e.g. juice dispensers) have previously been introduced into the enclosures for research purposes [62,63].

### 2.2. Data collection

The video recordings were collected throughout eight months during three separate field studies (June–August 2018, August–September 2015, July–September 2013), from 07.00 to 17.00 h to account for chimpanzees’ varying activity patterns throughout the day [64]. An observational approach [65] with focal-animal sampling [66] was employed, whereby each subject, as well as all individuals within 10 m of proximity to the focal animal, were recorded for 5 min using Panasonic HC v. 727 full HD, Sony CX405 Handycam full HD, and Sony Handycam DCR-TR v. 19E cameras. When there were no visible focal individuals during the observation period, ad libitum recordings were conducted. These recordings included all individuals within the observer’s visible range. The primary focus during ad libitum recordings was on individuals who had the least amount of collected video footage.

Across the three data collection periods, 79 h 20 min of observations were recorded (2018: 54 h 10 min, 2015: 18 h 5 min, 2013: 7 h 5 min). We then reviewed the collected footage for events with object actions produced by immature chimpanzees. Since not all videos from the 2013 field trip were included, the recordings were selected randomly and searched for all instances of such object actions, irrespective of object type or chimpanzee behaviour, to address the risk of selection bias. Data were only obtained from a single field study year for each subject to avoid uneven sampling of individuals across study years. Overall, a total of 15 h 15 min of recordings was used for this study’s analysis (infants: 8 h [67] and juveniles: 7 h 15 min [68]), the majority of which was extracted from videos collected in 2018 (19 subjects: colony 1, 2 and 3), followed by 2015 (10 subjects: colony 1 and 2), and 2013 (2 subjects: colony 4).

### 2.3. Behavioural coding of video recordings

Object actions (see [14]) were defined as instances when a subject used a physical object during a social interaction with a conspecific which could happen in two ways. First, when an object action was aimed towards a conspecific or resulted in physical contact through the object (e.g. touching, hitting and poking). The second, when the subject and their social partner were in close social proximity (<2 m) (e.g. walking or sitting together), showing directed face and/or engaged in an ongoing social interaction that involved an object. This included events which were part of or proceeded a social interaction where an object was either manipulated (e.g. shaking, waving and moving), acted upon without physical contact (e.g. peering at close distance) or used as substrates (e.g. ground and tree log) where it appeared to be selected for this action (e.g. stepping on and slapping).

The frequency of each action was coded with the onset at the start of the initial movement associated with the object action (e.g. an arm raise and extension preceding throwing). The conclusion of each action was indicated by either object release, change in social partner (e.g. leaving) or beginning of a subsequent object action. The apparent social partner (the recipient) was determined by proximity to the subject, face directedness and/or recent social engagement. We first assessed face directedness (e.g. whether the face was directed towards the social partner), followed by the subject’s most recent social interaction (e.g. having played with the social partner) and their subsequent behaviour (e.g. approaching the social partner) to determine the inferred recipient of each object action. To address pseudoreplication [69], object actions could comprise individual events produced either as a single unit or repeatedly as part of a rapid series. If an object action was repeated within 3 s of the previous action it was coded as belonging to the same series. If, however, the action involved a change in either an object or social partner, or followed 3 s after the preceding action, it was coded as a separate unit. To account for variability in temporal intervals used in primate gesture research, a 3 s interval was selected as a midpoint between the commonly used 1–5 s intervals [70,71]. Twenty-three types of object actions were observed in this study including: hitting, hitting threat, holding/carrying, moving, object in the mouth, peering, poking, presenting, pulling, pulling attempt, reshaping, shaking, slapping, spitting water, splashing, stepping, tapping, throwing around, throwing at, throwing threat, touching and waving (for definitions, see [14]).

For every object action, we coded the object type (natural versus artificial) and the subjects’ communicative expressions (vocalization, facial expression and face directedness). First, all 31 objects were classified into two categories: natural objects and artificial objects. Natural objects (17 items) referred to materials that naturally occur in the environment (e.g. leaves, sticks, branches, etc.) and excluded animals. Artificial objects (14 items) represented a variety of manufactured articles, including everyday use items (e.g. plastic containers, plastic bags, plastic tubes, etc.) as well as fragments of artificial objects (e.g. pieces of plastic and fabric scraps; see electronic supplementary material, table S2 for details). We then coded the subjects’ communicative expressions (their vocalizations and facial expressions) and face directedness that overlapped with at least a portion of the object action or occurred 2 s before or after the event [72]. Vocalizations (present versus absent) referred to 14 different audible sounds previously described in the vocal repertories of great apes [73,74]. These, for example, included whimpers, squeaks, laughter, hoots and grunts. Facial expressions (present versus absent) signified changes in the visible muscle movements of the face [75] in accordance with the previously described prototypical chimpanzee expressions. Specifically, the presence of a facial expression was coded if a chimpanzee exhibited a particularly expressive facial display, including pout faces, whimper faces, alert faces, bared teeth displays, scream faces, open mouth faces and pant hoot faces [76–78]. The absence of facial expression referred to the absence of expressive facial displays and was coded when the facial expression was neutral and relaxed (see electronic supplementary material, table S3 for descriptions of facial expressions).

Face directedness (directed versus non-directed) was also identified. The directed face was coded when the subject’s face was oriented towards the social partner and judged to be <45° of the conspecific with nothing impacting vision (as in [79]). This also included mutual face directedness. Face non-directed was coded when the subject’s face was oriented towards the conspecific and judged as oriented to be > 45° of a direct line to the centre of the social partner’s face or when the social partner was facing fully away from the subject. As in other studies (e.g. [32,80]), the response of the social partner to the subject’s object actions signified changes in the recipient’s behaviour (e.g. face directedness and movement) following the object action (<5 s). This was used to examine the level of responsiveness to object actions with natural and artificial objects among the social partners.

Supplemental coding included object selection and object recurrence. A subject’s object selection referred to the object type (natural versus artificial) that was selected to produce an object action when both natural and artificial objects were within the chimpanzee’s hand reach (i.e. <1 m) [51,59]. The social partner’s object selection referred to the object type (natural versus artificial) that was subsequently picked up by the social partner after being released by the subject. Lastly, the recurrence of artificial objects was examined with reference to instances when the same artificial object (e.g. same water bottle) was used by a subject as part of their object actions across two consecutive days (day 1 versus day 2).

Cohen’s kappa [81] was used to evaluate the intercoder reliability based on 30% of randomly selected coded events (66/166 focal recordings). High or very high levels of agreement [82] were found for all coding categories: object type (κ = 0.96), object actions (κ = 0.91), vocalizations (κ = 0.70), facial expressions (κ = 0.86) and face directedness (κ = 0.83).

### 2.4. Statistical analysis

We first addressed the use of objects by describing the total number of object actions produced by all 31 subjects during social interactions, and to which examined behaviours these related (i.e. facial expressions, vocalizations and visual attention). To assess the impact of object types (natural versus artificial) on the production of chimpanzees’ facial expressions, vocalizations and directed facing, we used three generalized linear mixed models (GLMM) with a binomial error structure. Model 1 estimated the effect of colony membership and an interaction effect (object type × age group) on whether the subject produced facial expression as part of the object action. At this stage, we also evaluated the models with and without an interaction term. The comparison revealed that the model including the interaction term (AIC: 971.19, BIC: 1007.0), fit the data significantly better than the model without an interaction term (AIC: 973.80, BIC: 1008.0; χ^2^(4) = 4.61, *p* = 0.03).

Model 2 estimated the effect of colony membership, object type and age group on whether the subject produced vocalization during an object action. Initially, object type and age group were included as an interaction term, but this resulted in a model convergence failure. The third and final model (model 3) estimated the effect of colony membership and an interaction effect (object type × age group) on whether the subject produced directed facing. Since we had multiple data points for each subject, the subject’s ID was used as a random factor in all models to account for the individual differences between the chimpanzees. An interaction effect in model 1 and model 3 helped to examine how the effect of object type (on facial expressions and face directedness) may differ depending on the subject’s age group (infant versus juvenile). In the initial designs, all models included the subject’s sex and colony membership as predictors. However, when both were included, the models failed to converge. It is likely that the models were too complex relative to the available data to estimate the model parameters with sufficient accuracy. We decided to exclude the sex predictor in favour of colony membership, as the models with colony predictor showed a better fit compared to those with sex predictor (see electronic supplementary material, table S1 for likelihood ratio test). In addition, there is some evidence suggesting that the examined chimpanzee colonies vary in their social climate and communicative behaviours [14,83]. All final models successfully converged, showed a superior fit against the null models (containing random factors only; see the electronic supplementary material) and were compared using the likelihood ratio test. All models were evaluated and met the assumptions of normality of residuals, homogeneity of variances and absence of multicollinearity (see the electronic supplementary material for corresponding values). Wald χ^2^ with confidence intervals (CI) of 95% were used to assess each parameter in the final models. Marginal *R* ^2^ values were calculated for the models [84]. No cases of missing data were reported. The analysis was carried out using RStudio v. 2023.03.0 + 386 (RStudio, Boston, MA, USA) using the ‘(g)lmer’ function (‘lme4’ and ‘optimix’ packages).

To examine object selection, object recurrence and the level of the social partner’s responsiveness we used nonparametric tests as the data were not normally distributed based on the Shapiro–Wilk normality test and visual inspection of the data. Percentages were used to account for interruptions that resulted from the subjects being out of view (e.g. by temporarily withdrawing into the forest) and were calculated based on frequencies. To examine the differences in the object selection and response of the social partner between natural and artificial objects, we used Wilcoxon signed-rank tests with a rank-biserial correlation coefficient to estimate the effect size [85,86]. Looking at object recurrence, we compared the differences in the percentage of communicative expressions (vocalizations + facial expressions) that involved the same artificial objects between day 1 and day 2. The analyses were conducted using SPSS Statistics 27 (IBM, Chicago, IL, USA) with *p* < 0.05 used to assess the significance and if needed Holm’s corrections were applied to control for family-wise error rate [87].

## 3. Results

A total of 731 object actions were coded, including 418 with natural objects (17 types) and 313 with artificial objects (14 types) (see electronic supplementary material, table S2 for frequencies and descriptions). The object actions may have been performed using the same objects, which was particularly the case for the artificial objects that were less common and restricted to a smaller selection. Of the observed object actions, 668 (91.38%) met at least one of the following criteria: (i) directed towards another; (ii) involved gazing at the social partner; or (iii) triggered a response in the social partner. Of the remaining 63 actions, 14 occurred as part of or immediately before/after a social interaction with the social partner (e.g. throwing a stick while running away from the social partner). The final 49 actions were produced in close proximity to the social partner (<1 m).

### 3.1. Facial expressions: age comparison for natural and artificial objects

All 31 chimpanzees in this study produced facial expressions in co-occurrence with object actions (see table 2). A full model (model 1) that predicted the occurrence of facial expressions (AIC: 971.19, BIC: 1008.0) fit the data significantly better than the null model (AIC: 994.35, BIC: 1003.5; χ^2^(6) = 35.15, *p* < 0.001). The proportion of marginal variance attributed to the fixed effects in model 1 was *R* ^2^ = 0.07.

**Table 2.** Total frequency (#) and mean percentage of facial expressions, vocalizations and directed facing produced with natural objects and artificial objects by infant and juvenile chimpanzees with the number of individuals producing these behaviours in parentheses (*N*).

	infants				juveniles			
	natural		artificial		natural		artificial	
	# (*N*)	mean% [s.d.]	# (*N*)	mean% [s.d.]	# (*N*)	mean% [s.d.]	# (*N*)	mean% [s.d.]
facial expressions	118 (13)	41.84 [17.89]	105 (13)	80.61 [18.05]	85 (17)	46.13 [23.70]	85 (17)	58.74 [30.07]
vocalizations	0 (0)	0 [0.0]	3 (3)	1.45 [3.24]	7 (5)	2.42 [4.44]	31 (8)	16.58 [20.74]
directed facing	189 (13)	72.32 [13.41]	148 (13)	94.27 [7.48]	152 (18)	75.40 [21.78]	124 (18)	78.25 [21.93]

**Figure 1.**
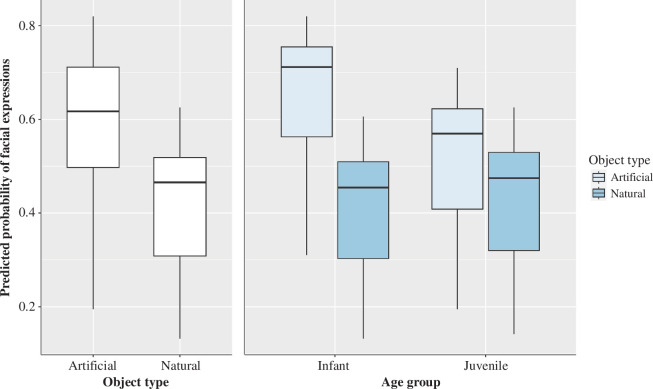
Main effects of object type that successfully predicted the occurrence of facial expressions. The light blue boxes represent artificial objects and the dark blue boxes natural objects. The thick horizontal lines depict medians and the thin lines maximum and minimum range values. The upper and lower quartiles are indicated by the box lengths. The vertical lines indicate 95% confidence intervals.

In the full model, a significant effect of object type and colony membership was found with facial expressions significantly less likely to occur during interactions with natural objects (estimate  ±  s.e. = −1.09  ±  0.24, *p* < 0.011) and among the members of colony 3 (estimate  ±  s.e. = −1.32  ±  0.57, *p* = 0.021). There was also a significant interaction between object type and chimpanzees’ age group (figure 1). Facial expressions were significantly more likely to occur among the juveniles when using natural objects, but not among the infants who produced more facial expressions with artificial objects (estimate  ±  s.e. = 0.70  ±  0.33, *p* = 0.032). All model values for the full model 1 are shown in table 3.

**Table 3.** Features of object type × age group and colony membership as predictors of chimpanzees’ facial expressions (*n* = 31).

predictors	*B* (s.e.)	95% Wald confidence interval for (*B*)		Wald χ^2^ (d.f.)	*z value*	*p-*value
		lower	upper			
(intercept)	1.09 (0.45)	0.21	1.97	5.96	2.44	0.015
object type: natural	−1.09 (0.24)	−1.55	−0.63	21.34	−4.62	0.000
age group: juvenile	−0.62 (0.31)	−1.22	−0.02	4.13	−2.03	0.042
juvenile × natural	0.7 (0.33)	0.06	1.35	4.58	2.14	0.032
colony: one	−0.25 (0.44)	−1.1	0.6	0.33	−0.57	0.566
colony: two	−0.04 (0.4)	−0.82	0.74	0.01	−0.11	0.920
colony: three	−1.32 (0.57)	−2.44	−0.2	5.29	−2.30	0.021

### 3.2. Vocalizations: age comparison for natural and artificial objects

The data of this study showed that 14 of the 31 chimpanzees vocalized during object actions (see table 3). A full model (model 2) that predicted the occurrence of vocalizations (AIC: 276.41, BIC: 308.58) fit the data significantly better than the null model (AIC: 328.61, BIC: 337.80; χ^2^(5) = 62.19, *p* < 0.001). The proportion of marginal variance attributed to the fixed effects in model 1 was *R* ^2^ = 0.96.

**Figure 2.**
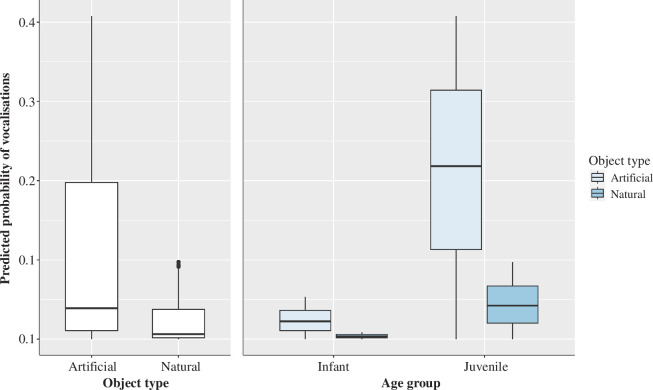
Main effects of object type that successfully predicted the occurrence of vocalizations. The light blue boxes represent artificial objects and the dark blue boxes natural objects. The thick horizontal lines depict medians and the thin lines maximum and minimum range values. The upper and lower quartiles are indicated by the box lengths. The vertical lines indicate 95% confidence intervals.

In the full model, a significant effect of object type was found with vocalizations significantly less likely to occur during interactions that involved natural objects (estimate  ±  s.e. = −1.85  ±  0.38, *p* < 0.001) (figure 2). There was also a significant effect of age group, where vocalizations were more likely to accompany object actions produced by juvenile chimpanzees (estimate  ±  s.e. = 2.50  ±  0.52, *p* <0.001). There was no significant effect of colony membership on the chimpanzee’s vocal activity. All model values for the full model 2 are shown in table 4.

**Table 4.** Features of object type, age group and colony membership as predictors of chimpanzees’ vocalizations (*n* = 31).

predictors	*B* (s.e.)	95% Wald confidence interval for (*B*)		Wald χ^2^ (d.f.)	*z value*	*p-*value
		lower	upper			
(intercept)	−2.98 (0.64)	−4.23	−1.73	21.71	−4.66	0.000
object type: natural	−1.85 (0.38)	−2.60	−1.11	24.02	−4.90	0.000
age group: juvenile	2.5 (0.52)	1.48	3.53	22.84	4.78	0.000
colony: one	−19.46 (110.42)	−235.88	196.96	0.03	−0.18	0.862
colony: two	−0.48 (0.42)	−1.31	0.35	1.30	−1.14	0.254
colony: three	−1.16 (0.73)	−2.59	0.27	2.53	−1.59	0.112

### 3.3. Visual attention and responsiveness

All 31 chimpanzees showed directed face during object actions (see table 3). A full model (model 3) that predicted the occurrence of directed face (AIC: 633.23, BIC: 669.99) fit the data significantly better than the null model (AIC: 647.94, BIC: 657.13; χ^2^(6) = 26.71, *p* < 0.001). The proportion of marginal variance attributed to the fixed effects in model 1 was *R* ^2^ = 0.12.

**Figure 3.**
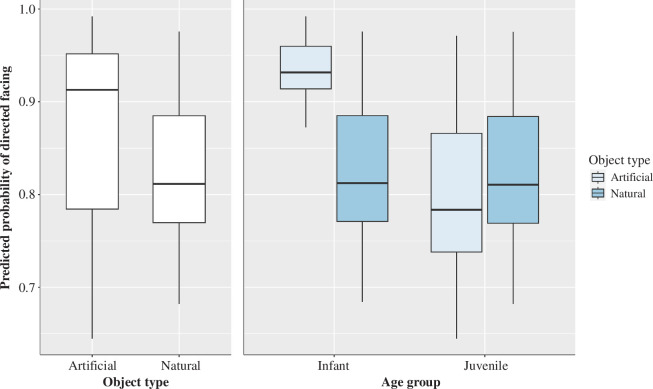
Main effects of object type and object type × age group interaction that successfully predicted the occurrence of directed facing. The light blue boxes represent artificial objects and the dark blue boxes natural objects. The thick horizontal lines depict medians, and the thin lines maximum and minimum range values. The upper and lower quartiles are indicated by the box lengths. The vertical lines indicate 95% confidence intervals.

In the full model, several significant effects were found with directed face significantly less likely to occur during actions with natural objects (estimate  ±  s.e. = 2.59  ±  0.60, *p* < 0.001) and when performed by juvenile chimpanzees (estimate  ±  s.e. = 1.65  ±  0.33, *p* < 0.001; see figure 3). However, there was also a significant interaction between object type and chimpanzees’ age group. Directed facing was significantly more likely to occur among the infants when using artificial objects, but more likely to occur when using natural objects among the juveniles (estimate  ±  s.e. = 1.65  ±  0.33, *p* < 0.001). The analysis showed a significant main effect associated with the chimpanzees’ colony membership. Specifically, it was found that compared to colony 4 directed facing during object actions was less likely to occur among the members of colony 1 (estimate  ±  s.e. = 1.65  ±  0.33, *p* < 0.001), colony 2 (estimate  ±  s.e. = 1.65  ±  0.33, *p* < 0.001) and colony 3 (estimate  ±  s.e. = 1.65  ±  0.33, *p* < 0.001). All model values for the full model 1 are shown in table 5.

**Table 5.** Features of object type × age group, and colony membership as predictors of directed facing (*n* = 31).

predictors	*B* (s.e.)	95% Wald confidence interval for (*B*)		Wald χ^2^ (d.f.)	*z value*	*p-*value
		lower	upper			
(intercept)	−1.15 (0.35)	−1.84	−0.45	10.51	−3.242	0.001
object type: natural	−1.33 (0.41)	−2.13	−0.52	10.47	−3.235	0.001
age group: juvenile	1.32 (0.46)	0.41	2.22	8.18	2.861	0.004
natural × juvenile	−2.04 (0.77)	−3.55	−0.54	7.07	−2.66	0.008
colony: one	−1.88 (0.79)	−3.43	−0.33	5.66	−2.379	0.017
colony: two	−2.45 (0.85)	−4.11	−0.79	8.33	−2.887	0.004
colony: three	−1.15 (0.35)	−1.84	−0.45	10.51	−3.242	0.001

We also compared the level of responsiveness to object actions with natural and artificial objects among the social partners. The analysis showed that the social partners responded more often to object actions that involved artificial objects than to those that involved natural objects (exact two-tailed Wilcoxon signed-rank test, *n* = 31 chimpanzees, *Z* = −3.02, *p* = 0.002).

### 3.4. Object selection and object recurrence

Due to the semi-wild nature of the enclosures, natural objects were always available for the chimpanzees to use while interacting. Out of the 731 reported object actions, 441 occurred when both natural and artificial objects were available (within visible range and excluding permanent fixtures, e.g. fences), and 290 occurred when only the natural objects were available, with no artificial objects within visible range at the time (see electronic supplementary material, table S2 for object types and frequencies). Object selection was measured in both the chimpanzee subjects and their social partners. When both natural and artificial objects were within the subjects’ hand reach (i.e. < 1 m), they selected artificial objects significantly more often than natural objects (exact two-tailed Wilcoxon signed-rank test, *n* = 31 chimpanzees: *Z* = −4.87, *p* < 0.001; chi-square test for association, χ^2^(1) = 399.75, *p* < 0.001). The majority of the subjects’ object actions that involved object retrieval, such as object pulling and pulling attempts, were produced with artificial objects (77.42%, 48 events), compared to natural objects (22.58%, 14 events). As for the social partner, when an object was released by the subject, artificial objects were significantly more often picked up by the social partner than natural objects (exact two-tailed Wilcoxon signed-rank test, *n* = 31 chimpanzees, *Z* = −3.07, *p* < 0.001, chi-square test for association, χ^2^(1) = 38.04, *p* < 0.001).

In addition, we examined the effect of object familiarity on chimpanzee behaviour by comparing interactions that featured the same artificial object across two observation points. The preliminary analysis into object recurrence showed that the chimpanzees who interacted with the same artificial object across two different days (i.e. day 1 versus day 2) showed no significant differences in their combined communicative expressions (facial expressions + vocalizations) (exact two-tailed Wilcoxon signed-rank test, *n* = 10 chimpanzees: *Z* = −0.10, *p* = 0.944).

## 4. Discussion

The present study tested the potential impact of objects used in social interactions on the communicative behaviour of immature sanctuary-living chimpanzees. The results revealed that the chimpanzees produced both facial expressions and vocalizations significantly more often when using artificial objects (e.g. plastic containers and cloths) compared to natural objects (e.g. sticks and stones) when interacting with their conspecifics. These findings provide the first empirical evidence that a selection of specific objects used in social interactions has the potential to promote communicative signalling early in chimpanzee ontogeny.

Interestingly, our main findings with the immature chimpanzees are in line with previous findings on human infants, where artificial objects (mostly toys) seem to promote vocal production [4,5] more than natural objects [6] so that they arguably play an important role in fostering communicative development [5,6,68]. Play with toys also seems to promote the expression of certain types of smiles, more than other social activities, such as book reading [3]. As a more frequent production of communicative behaviours is also likely to contribute to communicative development in primates [88], we similarly argue that social interactions with specific objects, in our case artificial objects used within a semi-natural setting, may promote communicative skills in chimpanzees. These findings thus suggest that the communicative behaviours of apes can be shaped by objects from early in development. It means that some types of objects may promote communication more than others in these individuals, comparably to the impact it has on children. Thus, the current study demonstrates a close relationship between objects and communication development from an evolutionary perspective.

As done in the human infant literature [4,5], it was important in this study to evaluate which factors may affect the communicative behaviours among the chimpanzees. One explanation is that the artificial objects used in the present study may have had more appealing physical features than natural objects that attract special interest and encourage social interactions. It is possible that the appeal of artificial objects could be related to their visual distinctiveness, a point that has also been made in the human literature [89,90]. Specifically, the distinctive features of artificial objects, which visibly stand out against the abundance of natural materials, are likely to generate such preference in chimpanzees.

In fact, our additional analysis suggests that the chimpanzee subjects showed a preference towards artificial objects, and when presented with an option to choose, artificial objects were selected significantly more often than natural objects. Given that chimpanzees can engage in communicative behaviours with objects simply for the sake of sharing attention [27], it is reasonable to assume that particularly appealing objects, such as the artificial objects used in the present study, could lead to more communication and social interactions. This explanation is further supported by previous research, where chimpanzees attend to object features [48,91,92] and appear to hold a preference towards objects that are distinctive in their appearance [51,93], where the suitability to be used as tools could also contribute to the object’s level of appeal [38,48].

It is, therefore, likely that the artificial objects *per se* were of more interest to the chimpanzees at the study site, including the social partners of our chimpanzee subjects. Thus, another explanation for why the subjects showed more communicative behaviours when using artificial objects compared to natural objects is that the use of the former received more attention from the social partners, which could further contribute to their overall appeal. This is supported by our results, in particular the preference of the social partners, who upon object release by the subject, had picked up artificial objects significantly more often than natural objects. Furthermore, our results also indicate that directed facing occurred more often when the chimpanzees were using artificial objects compared to natural objects during interactions with their social partners, but this pattern was only observed among the younger infant chimpanzees. While this form of visual attention in young primates has been much discussed as a form of information-seeking behaviour [40,52,53], their communicative behaviours are also known to change when they receive the attention of social partners [94,95]. Therefore, what may make an object appealing is the behavior of the social partner who might be interested in the specific object, i.e. the artificial objects in our study. Related to this, playful competitions over objects among young primates regularly involve obtaining and retaining the possession of particular materials [67,96,97]; for instance, young gorillas may hold up, show or throw objects to re-engage their social partners in play activities [67]. Specifically, it would be useful to examine the extent to which there may be socio-ecological factors affecting communication with natural and artificial objects, especially as such factors are known to have an impact on children [98–100].

In addition, our findings also suggest that artificial objects prompted more responses from the social partners than those involving natural materials. Among primates, vocalizations may be emitted as part of attention-getting behaviors [17,18,101] and during higher states of autonomic arousal [60]. It is, therefore, possible that the chimpanzees of this study produced vocalizations when interacting with artificial objects to attract the attention of the social partners, which elicited more responses, or due to arousal related to the attention that these objects appear to attract.

We also obtained interesting outcomes from a developmental perspective. The primary use of facial expressions with artificial objects as well as the occurrence of directed facing seem to be the outcome of the infants showing such behaviours, but not the juvenile chimpanzees of our study. The chimpanzee infants may have engaged in more directed facing perhaps to seek social engagement [102] or to seek information from the social partner [103]. This explanation is comparable with that found in human infants who appear to engage in more social gazing with their carers to seek approval or information about the attended objects [6,104]. As such, it is possible that among younger, less experienced chimpanzees, interactions with artificial objects may prompt more communicative expressions to attract the attention of others.

Interestingly, the juveniles of our study seemed to be the ones who predominantly vocalized when using artificial objects, although the findings on vocal activity of the infants, with only three vocal utterances, should be treated with caution. Nonetheless, the data on the juveniles suggest a different pattern in vocalizations and facial expressions when it comes to communicating while using artificial objects. It is possible that the communicative expressions of juveniles may stem from a higher state of autonomic arousal [105] when interacting with particularly appealing objects, such as artificial ones. This is akin to human children, whose vocal expressions can be influenced by arousal and objects [4,6,106], although their interest in objects is often shaped by additional factors. These factors may include the cultural significance of certain items, including their intended functions [107], as well as the child’s personal associations with objects based on past experiences [6]. This is particularly relevant given that objects frequently serve to establish shared intentionality and create joint experiences [35]. For instance, children may point to, show or offer objects as a means of initiating joint attention or cooperative play. Therefore, the social interactions centred around objects likely contribute to their overall appeal. Consequently, it would be important to explore the potential cultural relevance of specific objects among chimpanzees, as well as their object associations, to understand how these factors may influence the perceived appeal of objects.

On reflection, it is, however, important to be cautious when it comes to generalizing the impact of artificial objects on communication for other chimpanzees. The chimpanzees of our study most likely were less familiar with the artificial objects than the natural objects, because the former were only occasionally present (23 artificial objects were found altogether during a three month field trip in 2018) and often stayed no more than one day in the enclosures. In contrast, natural objects were always available for interaction due to the semi-wild environment in which the chimpanzees live. While the chimpanzees did not seem to differ in their communicative behaviours when they were first observed interacting with an artificial object compared to the later episode, we cannot, with confidence, rule out the effect of object familiarity. The artificial objects used by the chimpanzees of this study were also not new to them and carried many markers of prior handling, but again we cannot comment on the extent to which these were used by the specific chimpanzees that we observed.

Previous research suggests that the motivation to interact with an object is mainly motivated by one’s familiarity, then the increased habituation following exploration may contribute to a decline in interest [108]. Yet, it is possible that certain objects that hold enough appeal will continue to generate interest [28], whereas for some others, this effect may wane as a function of exposure [30]. Although we might expect some differences in familiarity between artificial and natural objects (primarily due to the less consistent presence of the artificial objects), in our study, the chimpanzees were already relatively accustomed to interacting with similar artificial objects, which were not novel but rather common types that had previously been present in their enclosures. These objects bore signs of wear and tear from prior handling, indicating they were not unfamiliar stimuli likely to elicit heightened communicative behaviours due to novelty. This suggests that the observed increase in the communicative expressions with artificial objects indicates a distinct influence of these objects on communicative behaviour. Our cautious consideration of the differences in familiarity between natural and artificial objects serves to acknowledge the potential influence of familiarity on behaviour without suggesting that the artificial objects were novel or highly unfamiliar to the chimpanzees. In the context of the present study, more longitudinal research would be needed to ascertain the extent to which object familiarity impacted the communicative behaviour of the chimpanzees.

Altogether, these findings provide evidence that specific types of objects are more likely to prompt communicative expressiveness in nonhuman primates. Therefore, the current study provides first empirical evidence that the appeal of artificial objects is likely to contribute to increased communicative behaviours in chimpanzees. It also highlights a close relationship between objects and communication development from an evolutionary perspective. Admittedly, it is unclear whether this effect stems from the features of artificial objects, the attention these objects seem to attract from others or perhaps the familiarity level. However, long before they can speak, human infants sense that artefacts were made by agents with goals and intentions and that the intention of the creator defines the affordances of artefacts [109,110]. They are sensitive to the communicative cues that convey different types of cultural knowledge, including information about artefact kinds and their specific functions that are often cognitively opaque [111]. Consequently, our main findings are in line with previous accounts of language evolution and tool use, which suggests that vocal language was adapted to facilitate situational information sharing, including that involved in tool use [10], and may consequently had been implicated in language evolution [112–114]. Given that chimpanzees are the most proficient nonhuman tool users [115], with the largest animal tool-use repertoire, these findings suggest that the use of objects in social interactions may have helped to promote facial and vocal expressions earlier in human evolution than previously thought.

## Data Availability

Data included in the supplementary materials [116].
